# Multi-protocol exploratory treatment for metastatic castration-resistant prostate cancer with BRCA mutations: a case report and literature review

**DOI:** 10.3389/fonc.2023.1177941

**Published:** 2023-05-10

**Authors:** Dongsheng Zhao, Wen Su, Liang Zeng, Guoqian Hu, Jin Tang

**Affiliations:** Department of Urology, The Third Xiangya Hospital, Central South University, Changsha, Hunan, China

**Keywords:** prostate cancer, BRCA1 gene mutation, AVPC, mCRPC, PD-1

## Abstract

Most patients with metastatic hormone sensitive prostate cancer will progress to metastatic castration-resistant prostate cancer (mCRPC); Finding a highly effective, safe treatment with low recurrence rate has important clinical implications. Herein, we present a case of a 65-year-old man with castration-resistant prostate cancer treated by multi-protocol exploration. Magnetic resonance imaging (MRI) revealed prostate cancer invading the bladder, seminal vesicle glands, and peritoneum with pelvic lymph node metastasis. Transrectal B ultrasound puncture of prostate tissue was performed, and the pathological diagnosis was prostatic adenocarcinoma. CTC (Circulating tumor cell) gene test was performed in peripheral blood, and the result showed BRCA1 gene mutation. The patient died of tumor complications after trying docetaxel combined with cisplatin chemotherapy, PARP inhibitor (nilaparib), PD-1 inhibitor (tislelizumab) and other treatments. This patient showed that the selection of an individualized combination chemotherapy regimen based on genetic testing results benefited the patient’s tumor control. When choosing a treatment regimen, problems such as failure to respond to re-chemotherapy and resistance to nilaparib may lead to deterioration of the condition.

## Introduction

About 81% of the newly diagnosed prostate cancer cases in the United States are clinically localized, while most of the newly diagnosed prostate cancer cases in China are locally advanced or extensively metastatic at the time of diagnosis. Patients in this period cannot receive radical local treatment, and can only receive endocrine therapy, chemotherapy, immunotherapy, etc. Androgen deprivation therapy (ADT) can achieve remission in most patients in a short period of time, but almost all patients eventually develop metastatic castration-resistant prostate cancer (mCRPC). Recently, docetaxel, CYP17A1 inhibitor abiraterone and androgen receptor inhibitor enzalutamide have become the first line treatment for CRPC. In addition, immunosuppressive therapy and radium-223 are the research hotspots of CRPC. However, there are some problems in CRPC patients, such as gene mutation amplification, PD-1/PD-L1 immune target, AKR1C3 expression, AR-V7 and other AR splice variants. Therefore, in clinical work, the treatment of CRPC patients should be precise and individualized. It is necessary to combine the results of gene sequencing and molecular detection to accurately select individualized targeted therapy drugs for CRPC patients to maximize the benefit of patients. In this case, the patient with the BRCA mutation rapidly developed AVPC (aggressive variant prostate cancer) within six months of receiving endocrine therapy and died of complications after trying various regimens to no avail.

## Case presentation

**Figure 1:**
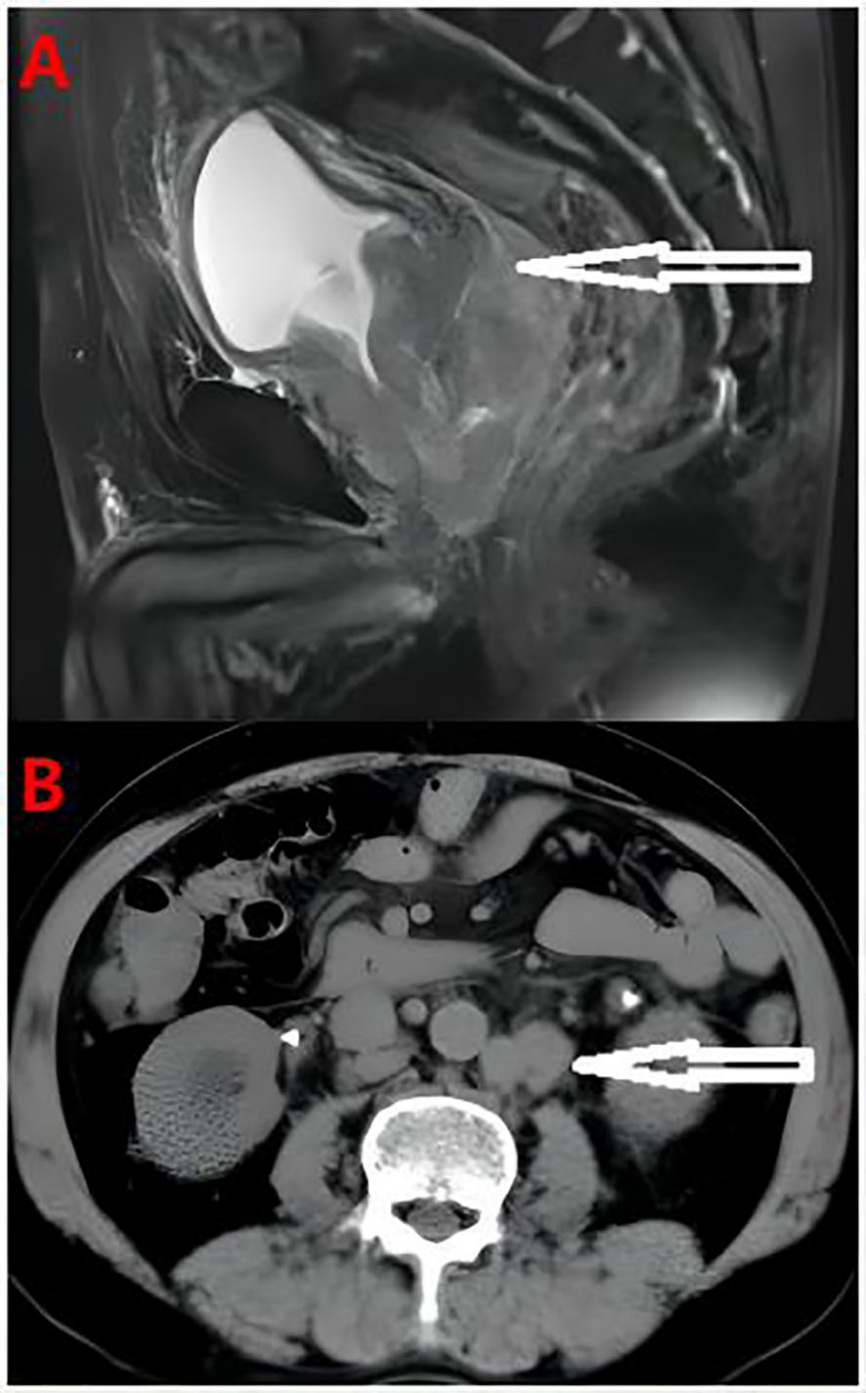
**(A)** Pelvic MRI Prostate cancer with bladder, bilateral seminal vesicle gland and peritoneum involvement, bilateral ureter and sigmoid colon involvement, secondary pelvic lymph node metastasis. **(B)** The abdominal computed tomography showed the tumor involved retroperitoneal lymph nodes.

A 65-year-old male patient was admitted due to “frequent urination, urgent urination with dysuria for more than 2 years” on August 15, 2019. Digital rectal examination showed grade III enlargement of the prostate, firm consistency, disappearance of the central sulcus, smooth surface, no palpable nodules, and no palpable bilateral seminal vesicles. The patient had no family history of prostate cancer. Total prostate specific antigen (TPSA) was 14.83 ng/ml, and magnetic resonance imaging (MRI) showed a prostate volume of 5.4 cm×7.6 cm×5.0 cm, considering prostate cancer with bladder, bilateral seminal vesicle, and peritoneal involvement, bilateral ureteral and sigmoid involvement, secondary pelvic lymph node metastasis, bilateral ureteral dilatation, and bilateral hydronephrosis (**Figure 1A**). Creatinine is 254 µmol/l, transrectal B-ultrasound-guided prostate biopsy, pathological diagnosis of prostate adenocarcinoma, Gleason score 5 + 5 = 10 points; immunohistochemistry: HCK (-), P63 (-), P504s (partial +), PSMA (+), PSA (-), NKX3.1 (+ +), AR (+ +), CgA (-), Syn (-), ki67 (60%), CD56 (-). Bone scan showed no metabolic abnormalities in any bone throughout the body.

**Figure 2:**
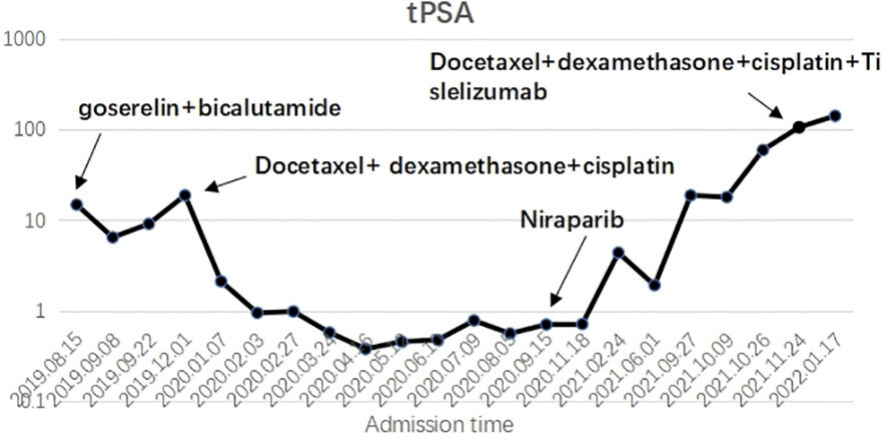
X axis: Admission time; Y axis: Blood tPSA ; The trend of tPSA changes after taking various methods during the treatment.

During the treatment and efficacy, the patient was diagnosed with metastatic hormone-sensitive prostate cancer (T4N1M1c) according to the examination data, and was given goserelin (once a month) combined with bicalutamide 50 mg (once a day) endocrine therapy. Blood tPSA was 6.48 ng/ml (**Figure 2**) at 2 weeks and 9.12 ng/ml at 4 weeks of treatment, and tPSA fluctuated significantly. On September 22, 2019, serum creatinine increased to 844 μmol/l, reaching uremia level. On September 24, 2019, the patient underwent bladder mass resection under plasmakinetic endoscopy + double ureteroscopy + double DJ catheter placement. Pathological examination of the intravesical mass showed prostate cancer invading the bladder. Creatinine decreased to 326 µmol/l 1 week after surgery. On December 1, 2019, blood tPSA was 16.07 ng/ml and creatinine increased to 746 µmol/l again. Renal dynamic imaging showed severely impaired bilateral renal function, right renal function better than the left, retroperitoneal lymph node metastases progressed, and compression of both ureters (**Figure 1B**). On December 2, 2019, percutaneous nephrostomy of the right side was performed, and creatinine decreased to 135 µmol/l.

**Figure 3:**
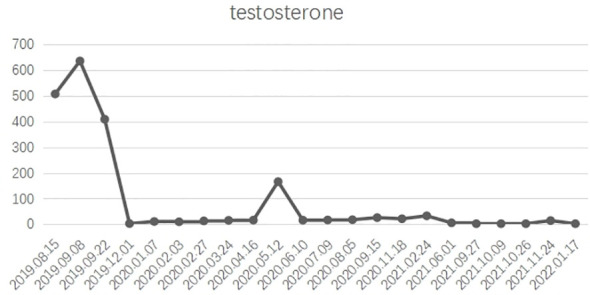
X axis: Admission time; Y axis: testosterone ; In general, castrate levels of testosterone were maintained after treatment.

**Figure 4:**
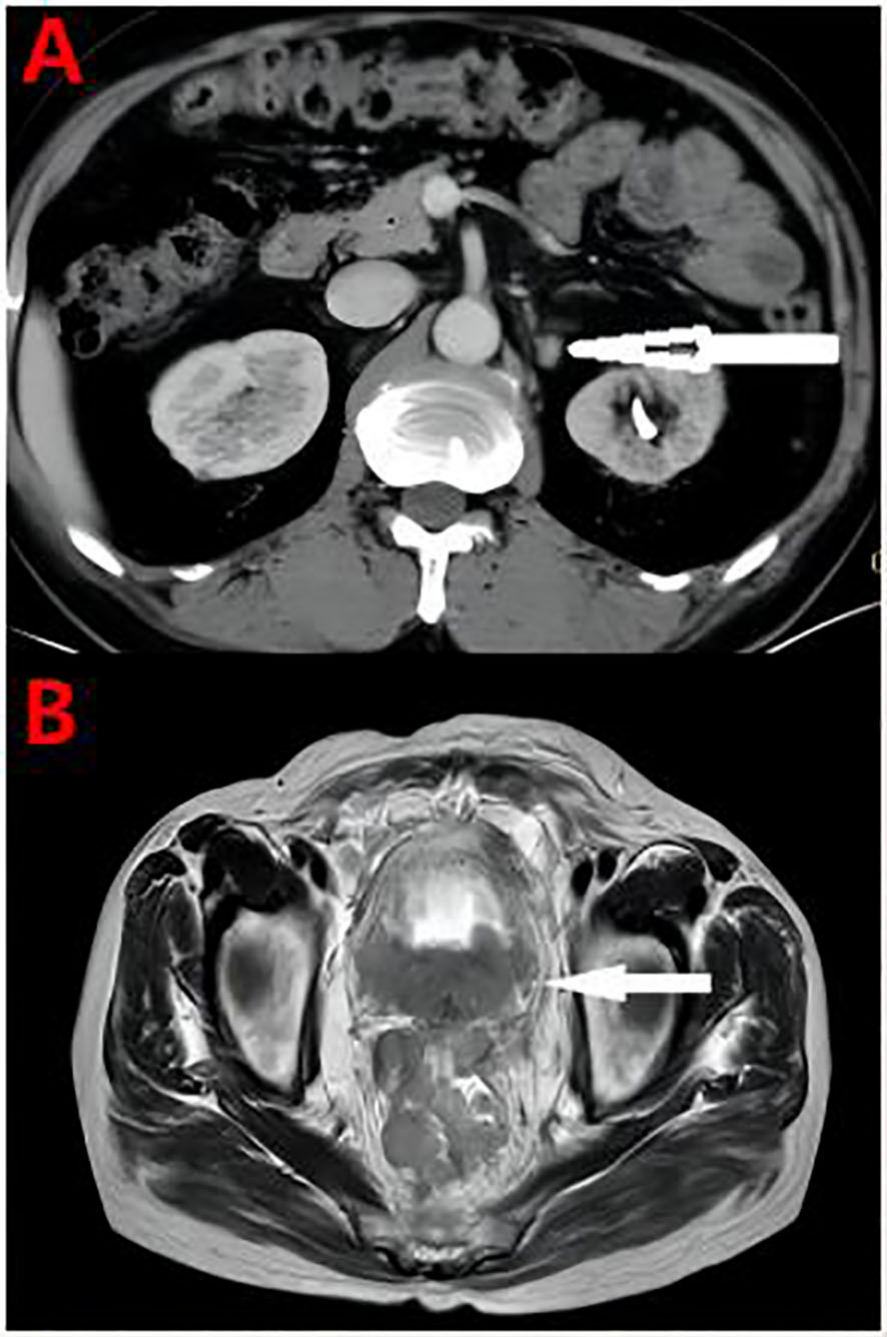
**(A)** At the end of the chemotherapy cycle, CT showed significant reduction of retroperitoneal lymph nodes. **(B)** Pelvic MRI Pelvic organ involvement and multiple pelvic lymph node metastasis were more advanced than before.

Overall, the patient had testosterone less than 2.5 ng/dl and maintained castrate levels (**Figure 3**), but both tPSA and imaging studies suggested disease progression. Patients were confirmed to enter castration-resistant prostate cancer (mCRPC) stage. CTC gene test was performed in peripheral blood, and the result showed BRCA1 gene mutation. Docetaxel + cisplatin + prednisone (5 mg bid) regimen was administered for chemotherapy every 4 weeks, and blood tPSA decreased to 0.988 ng/ml as assessed on February 27, 2020-27 after 4 times of chemotherapy. Repeated whole abdominal CT showed no progression. Six cycles of chemotherapy were continued, during which the patient ‘s blood PSA level was maintained at around 0.5 ng/ml. At the end of 10 cycles of chemotherapy, CT showed that retroperitoneal lymph nodes basically disappeared (**Figure 4A**), tPSA decreased to 0.564 ng/ml, and blood testosterone was < 2.5 ng/dl, and the DJ tube and right nephrostomy tube were removed.

On September 15, 2020, the patient began to receive Nilaparib 200 mg (qd) + Goserelin 3.6 mg (qm). During 1-year follow-up, blood tPSA fluctuated between 1.92-5.04 ng/ml and creatinine fluctuated between 120-136 µmol/l.On September 27, 2021, reexamination showed serum tPSA 18.79 ng/ml and creatinine 646µmol/l. CT showed aggravated right hydronephrosis due to progression of the right lower ureteral lesion. Right nephrostomy was performed again, and creatinine gradually decreased to 150 µmol/l after surgery, and nilaparib 200 mg + goserelin 3.6 mg was continued after full communication with the patient and his family. On November 23, 2021, re-examination showed serum tPSA 59.24 ng/ml and creatinine 159 μmol/l. CT and MRI showed prostate lesions progressed than before, and multiple intrahepatic metastases newly developed, which were considered as prostate cancer progression.The patient was given Docetaxel + Cisplatin + Prednisone 5 mg bid for chemotherapy. On December 24, 2021, blood tPSA 105.5 ng/ml and creatinine 143 µmol/l were reexamined, and CTC gene detection in peripheral blood was performed again: TP53, BRCA1, and BRCA2 gene mutations. After discussion by MDT, tislelizumab was added on the basis of chemotherapy regimen. After 5 times of chemotherapy, blood tPSA is 279.2 ng/ml and creatinine is 155 µmol/l. MRI showed pelvic organ involvement and multiple pelvic lymph node metastases progressed than before (**Figure 4B**).The patient refused to receive chemotherapy again and chose oral abiraterone + prednisone regimen. On May 21, 2022, the patient died of systemic multiple organ failure caused by prostate cancer.

At different time points in the development of the disease, timely intervention and treatment are carried out through different treatment strategies.

## Discussion

The efficacy of primary chemotherapy with platinum combined with paclitaxel in this patient was significant. Most patients with metastatic hormone-sensitive prostate cancer progress to metastatic castration-resistant prostate cancer, or mCRPC. Platinum chemotherapy may be an effective regimen for prostate cancer patients with BRCA mutations. The patient had the following characteristics: Gleason score 5 + 5, high malignancy, high primary tumor burden, relatively low tPSA at first, and very short response time to ADT, rapidly entering the mCRPC stage. According to these characteristics, this patient could be diagnosed with aggressive variant prostate cancer (1);Docetaxel-based chemotherapy is the standard chemotherapy regimen for mCRPC. The results of genetic testing (2) in this patient suggested BRCA1 gene mutation and sensitivity to platinum agents (3), and platinum therapy was associated with relevant antitumor activity in a biomarker-positive population of advanced prostate cancer patients with abnormal DNA repair genes according to Sabine Schmid et al. (4). Platinum is a kind of cell cycle-specific drugs, which exert anti-tumor activity by forming covalent compounds with cellular DNA to cause DNA damage. Platinums and taxanes are often combined in cancer therapy and several studies in mCRPC patients have assessed the effect of carboplatin or cisplatin either in combination with taxanes or as monotherapy, but results have been inconclusive. Multiple small phase I and II trials have shown the safety and efficacy of taxane-platinum combinations in patients with mPC, although some of the combinations resulted in significant toxicity. During 10 cycles of docetaxel + cisplatin chemotherapy (5), the PSA level was maintained below 1 ng/ml, the retroperitoneal lymph nodes were significantly reduced, the ureteral obstruction was significantly relieved, and the renal function was improved than before. It is shown that individualized combination chemotherapy based on genetic testing results is beneficial for tumor control.

After the end of treatment with docetaxel + cisplatin chemotherapy regimen, the patient was treated with PARP inhibitor nilaparib combined with endocrine therapy. Some studies (6) have reported that nilaparib can be used as maintenance therapy for malignant tumors after achieving complete or partial remission with first-line platinum-based chemotherapy. This patient was reexamined after 9 months of nilaparib maintenance therapy, blood tPSA 1.92 ng/ml, and PSA levels did not decrease over the following 3 months. Combined with imaging progression and deterioration of renal function, it was considered that the disease developed again due to nilaparib resistance, so docetaxel + cisplatin chemotherapy regimen was given again. As a novel targeted drug, PARP inhibitor nilaparib is one of the precise and individualized exploratory treatment options for advanced prostate cancer, but there are problems such as sensitivity and drug resistance in a small proportion of patients.

The patient had a poor response to a second platinum-based combined with violacetol chemotherapy, and docetaxel chemotherapy can be readministered in patients who have previously received docetaxel and respond to treatment, that is, docetaxel rechallenge (7). In this patient, 14 months after the end of docetaxel + cisplatin chemotherapy regimen, genetic diagnosis results showed TP53, BRCA1 and BRCA2 gene mutations, and docetaxel + cisplatin chemotherapy was given again to cope with disease progression (8). However, after the application of chemotherapy in this patient, the disease still progressed, suggesting a poor response for docetaxel rechallenge.

According to Brown, J.R et al. (9), tirelizumab (10) is a novel humanized IgG-4 programmed death receptor 1(PD-1) inhibitor. For PD-L1-positive urothelial cancer patients who are not eligible for cisplatin treatment, immunotherapy with tislelizumab has recently become an effective treatment. Continued use of tirelizumab may have benefited this patient, but follow-up was not possible due to her subsequent refusal of chemotherapy and further systematic treatment.

## Conclusion

This patient had significant efficacy after primary chemotherapy with platinum combined with paclitaxel, and failed maintenance therapy due to resistance or insensitivity to PARP inhibitor nilaparib; after disease progression, platinum combined with paclitaxel chemotherapy was ineffective again, and the outcome of tentative treatment with PD-1 inhibitor tislelizumab was unknown. Through the treatment process of this patient (**Table 1**), we can recognize that for metastatic castration-resistant prostate cancer patients with BRCA 1 gene mutation, seeking individualized precise treatment plan based on genetic testing results helps to control tumor progression early. In addition, the possible unique biological basis of AVPC remains to be clarified.

**Table 1:** Multiple treatment strategies and causes.

Date	TPSA ng/ml	Testos-teron-e ng/dl	Treatment strategies and Reasons
2019-08-15	14.83	507.60	goserelin + bicalutamide(conventional antiandrogen therapy)
2019-12-01	16.07	<2.50	docetaxel + cisplatin + prednisone(Rapidly entering the mCRPC stage, the high tumor burden and the results of genetic testing were considered,taxanol combined with platinum was chosen for treatment.)
2020-09-15	0.56	<2.50	nilaparib + goserelin(After achieving complete or partial remission with first-line platinum-based chemotherapy.Nilaparib can be used as maintenance therapy)
2021-11-23	59.24	<2.50	docetaxel+cisplatin+prednisone(Docetaxel chemotherapy can be readministered in patients who have previously received docetaxel and respond to treatment, that is, docetaxel rechallenge.)
2021-12-24	105.50	<2.50	docetaxel + cisplatin + prednisone +tislelizumab( On the basis of the results of repeat genetic testing, we elected to add a PD1-receptor inhibitor(tislelizumab).)
2022-01-07	279.20	<2.50	abiraterone + prednisone(The patient refused to receive chemotherapy again.)

## Data availability statement

The raw data supporting the conclusions of this article will be made available by the authors, without undue reservation.

## Ethics statement

Written informed consent was obtained from the individual(s) for the publication of any potentially identifiable images or data included in this article.

## Author contributions

JT: Revising the manuscript critically for important intellectual content. DZ: Drafting the manuscript. LZ, WS, and GH: Data collecting and sorting. All authors contributed to the article and approved the submitted version.
